# Trends, Patient and Prescriber Characteristics in Gabapentinoid Use in a Sample of United States Ambulatory Care Visits from 2003 to 2016

**DOI:** 10.3390/jcm9010083

**Published:** 2019-12-29

**Authors:** Lili Zhou, Sandipan Bhattacharjee, C. Kent Kwoh, Patrick J. Tighe, Daniel C. Malone, Marion Slack, Debbie L. Wilson, Joshua D. Brown, Wei-Hsuan Lo-Ciganic

**Affiliations:** 1 Department of Pharmacy, Practice and Science, College of Pharmacy, University of Arizona, Tucson, AZ 85721, USA; lilizhou@pharmacy.arizona.edu (L.Z.); bhattacharjee@pharmacy.arizona.edu (S.B.); slack@pharmacy.arizona.edu (M.S.); 2 University of Arizona Arthritis Center, University of Arizona College of Medicine, Tucson, AZ 85724, USA; CKwoh@arthritis.arizona.edu; 3 Department of Medicine, Division of Rheumatology, University of Arizona College of Medicine, Tucson, AZ 85724, USA; 4 Department of Anesthesiology, College of Medicine, University of Florida, Gainesville, FL 32610, USA; PTighe@ufl.edu; 5 Department of Pharmacotherapy, College of Pharmacy, University of Utah, Salt Lake City, UT 84112,USA; Dan.Malone@utah.edu; 6 Department of Pharmaceutical Outcomes and Policy, College of Pharmacy, University of Florida, Gainesville, FL 32610, USA; debbie.wilson@ufl.edu (D.L.W.); joshua.brown@ufl.edu (J.D.B.); 7 Center for Drug Evaluation and Safety, College of Pharmacy, University of Florida, Gainesville, FL 32610, USA

**Keywords:** gabapentinoid, gabapentin, pregabalin, ambulatory care visits, patient and prescriber characteristics, trend, approved indication, off-label use

## Abstract

Increasing gabapentinoid use has raised concerns of misuse and abuse in the United States (US). Little is known about the characteristics of gabapentinoid use in general clinical practice over time. This cross-sectional study used data from the National Ambulatory Medical Care Survey. We examined the trends of patient and prescriber characteristics and the diagnoses associated with US ambulatory care visits involving gabapentinoids for adult visits from 2003 to 2016. Using multivariable logistic regression, we estimated the adjusted proportion of gabapentinoid-involved visits among all visits and tested for trend significance. Among the weighted estimate of 260.1 million gabapentinoid-involved visits (aged 18–64 years: 61.8%; female: 61.9%; white: 85.5%), the adjusted annual proportion of gabapentinoid-involved visits nearly quadrupled from 2003 to 2016 (9.1 to 34.9 per 1000 visits; *P_trend_* < 0.0001), driven mainly by gabapentin. Nearly half had concurrent use with opioids (32.9%) or benzodiazepines (15.3%). Primary care physicians (45.8%), neurologists (8.2%), surgeons (6.2%), and psychiatrists (4.8%) prescribed two-thirds of the gabapentinoids. Most (96.6%) of the gabapentinoid visits did not have an approved indication for gabapentinoids among the first three diagnoses. Among US ambulatory care visits from 2003 to 2016, gabapentinoid use increased substantially, commonly prescribed by primary care physicians.

## 1. Introduction

Approximately one-third of adults in the United States (US) have at least one chronic pain condition and seek care primarily in ambulatory care settings [1,2]. In response to the misuse of and addiction to prescription opioids in the US, states, payers, and healthcare systems have implemented numerous laws, regulations, and policies to combat the opioid epidemic in the past few years [3]. However, using non-opioid analgesics or multimodal analgesia to ensure appropriate pain management while minimizing the risk of adverse outcomes of medications imposes challenges to healthcare providers.

A study of Medical Expenditure Panel Survey (MEPS) data reported that individuals using gabapentinoids tripled from 2002 to 2015 in the US [4]. Similarly, a UK study of primary care medical records found the rate of patients newly treated with gabapentinoids tripled from 2007 to 2017 [5]. This increasing use has raised safety concerns of misuse and abuse of gabapentinoids, especially among individuals with opioid use disorder [6,7]. Motivations for the misuse or abuse of gabapentinoids include recreational use, substitution for other drugs, and addiction, among others [7]. To better inform interventions and policies in general clinical practice, further investigation of the patient, prescriber and visit characteristics, and primary diagnoses associated with gabapentinoids in US ambulatory care settings, where chronic pain is primarily managed, is needed. We examined the trends of patient and prescriber characteristics and the primary diagnoses associated with gabapentinoids among US ambulatory care visits from 2003 to 2016.

## 2. Experimental Section

### 2.1. Study Design, Setting, and Cohort

This cross-sectional study used data from a national probability sample survey, the National Ambulatory Medical Care Survey (NAMCS). The NAMCS is a nationally representative sample of office visits reported by non-federally employed, office-based physicians, including hospital- and non-hospital-employed physicians selected from the master files of the American Medical Association (AMA) and the American Osteopathic Association (AOA) [8]. The NAMCS sample selection criteria exclude physicians in anesthesiology, pathology, and radiology specialties. The NAMCS collects information including patients’ demographics, diagnoses, prescription and over-the-counter drugs, prescriber specialties, and type of insurance coverage. It collects a random sample of approximately 30 visits annually from a 1-week reporting period from each participating physician. The medication data include medications administered, ordered, continued, or supplied during each visit. The NAMCS increased the number of medications recorded over time from eight medications collected in 2003 to 2011, to 10 medications in 2012 and 2013, and up to 30 medications in 2014.

Our analytical sample included all adult (age ≥18 years) patient visits in which a gabapentinoid was administered, ordered, continued, or supplied (hereafter referred to as gabapentinoid-involved visits) from 2003 to 2016. We used the Multum Lexicon Plus^®^ system to identify the medications of interest (Table S1). To make the findings comparable across years, our primary analysis included the first eight medications listed for each reported visit, similar to the approaches used in the prior literature [9,10]. This study used publicly available de-identified data, and the University of Arizona Institutional Review Board deemed its human subjects exempt from review.

### 2.2. Patient, Prescriber and Visit Characteristics

Patient characteristics were examined among all gabapentinoid-involved visits in the NAMCS data from 2003 to 2016, including age (18 to 64 or ≥65 years), sex, race/ethnicity (white or non-white), smoking status (current or former/non-smoker), insurance coverage status (government insurance—Medicare, Medicaid, children’s health insurance program, or other state-based programs; commercial insurance; or others), and the major visit reason (chronic problems/routine check-up or other). Visits with concurrent use of gabapentinoids with opioids and benzodiazepines (Table S1) were categorized as concurrent use with opioids, concurrent use with benzodiazepines, or concurrent use with both. Given that the number of chronic conditions was collected starting in 2005, a variable of ≥2 chronic conditions was created from each patient visit from 2005 to 2016. The number of chronic conditions in the NAMCS is a separate variable from the diagnosis variables [8].

Variables of prescriber characteristics included physician specialty, geographic region (Northeast, Midwest, South, or West), and urbanicity (metropolitan or non-metropolitan) of practice locations. The NAMCS categorizes physician specialties into 14 categories: general and family practice, internal medicine, pediatrics, general surgery, obstetrics and gynecology, orthopedic surgery, cardiovascular diseases, dermatology, urology, psychiatry, neurology, ophthalmology, otolaryngology, and others. Based on the data distribution and clinical knowledge of specialties most commonly prescribing gabapentinoids, for each patient visit, we created a physician specialty variable categorized as primary care (including general/family practice and internal medicine) and surgery (including general surgery and orthopedic surgery), psychiatry, neurology, or others.

To further characterize the gabapentinoid-involved visits, we measured whether or not one of the first three physician-reported diagnoses associated with the visit was for an approved gabapentinoid indication. The physicians chose the diagnoses they reported, with the first diagnosis designated as the “primary” diagnosis, and the subsequent diagnoses designated as “other” diagnoses [8]. The physicians could include chronic conditions in the diagnoses if the conditions were related to the visit. NAMCS collected up to three International Classification of Diseases, Ninth Revision, Clinical Modification (ICD-9-CM) diagnoses for each patient visit from 2003 to 2013 and increased to five ICD-9-CM codes starting 2014. In 2016, NAMCS started using ICD-10-CM codes. Similar to our measurement of medications, we only used the first three physician-reported ICD-9-CM/ICD-10-CM codes listed to identify visits involving FDA-approved indications for gabapentinoid use, including partial seizures, postherpetic neuralgia, restless legs syndrome (gabapentin only), diabetic peripheral neuropathy (pregabalin only), fibromyalgia (pregabalin only), and neuropathic pain associated with spinal cord injury (pregabalin only) from 2003 to 2016 (Table S2).

### 2.3. Statistical Analysis

Our analyses included two steps. First, among all adult ambulatory care visits, we estimated the national annual number and proportions of visits involving gabapentinoids (overall, and by gabapentin and pregabalin), opioids, and benzodiazepines. The complex survey design of NAMCS was adjusted to obtain national-level data using survey procedures (SURVEYFREQ and SURVEYLOGISTIC) in SAS version 9.4 (SAS Institute Inc., Cary, NC, USA). Since each record can represent thousands of visits, the NAMCS recommends that unweighted numbers should be used only to determine the number of sample cases [11]. When the unweighted number was less than 30 or the relative standard error was greater than 30%, we only reported unweighted numbers due to reliability concerns based on NAMCS’ recommendation. The weighted data produce annual national estimates. We then estimated the adjusted annual proportion of gabapentinoid-involved visits among all adult ambulatory visits with marginal standardization for patient and prescriber characteristics and tested the trend significance using multivariable logistic regression. Second, for the adult ambulatory visits involving gabapentinoids, we examined their patient and prescriber characteristics and the association of an appropriate diagnosis. We also compared the characteristic differences between gabapentin and pregabalin visits using standardized mean difference (SMD), wherein SMD > 0.1 was considered as having non-negligible differences [12].

We conducted stratification analyses to examine whether any differences exist in the trends of gabapentinoid use by the patient and prescriber characteristics described previously. A sensitivity analysis was conducted to include all medications collected in the NAMCS each year (i.e., 8 medications for years prior to 2012, 10 medications in 2012 and 2013, and 30 medications starting in 2014).

## 3. Results

Characteristics of gabapentinoid visits were similar across years. Among the overall weighted estimate of 260.1 million visits involving gabapentinoids from 2003 to 2016, 61.8% of the visits were from individuals aged 18 to 64 years, 61.9% were female, and 85.5% were white (Table 1). Gabapentinoid-involved visits were primarily from individuals having a governmental insurance (52.3%) and ≥2 chronic conditions (61.3%). Among the overall weighted estimate of 260.1 million ambulatory visits involving gabapentinoids, 2.5 million used both gabapentin and pregabalin.

**Figure 1:**
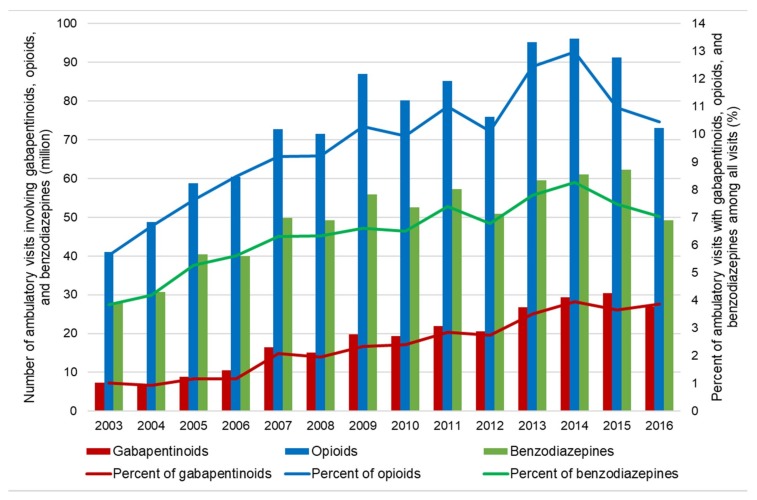
Trends in use of gabapentinoids, opioids, and benzodiazepines in the US ambulatory care settings: 2003–2016 National Ambulatory Medical Care Survey (NAMCS).

**Figure 2:**
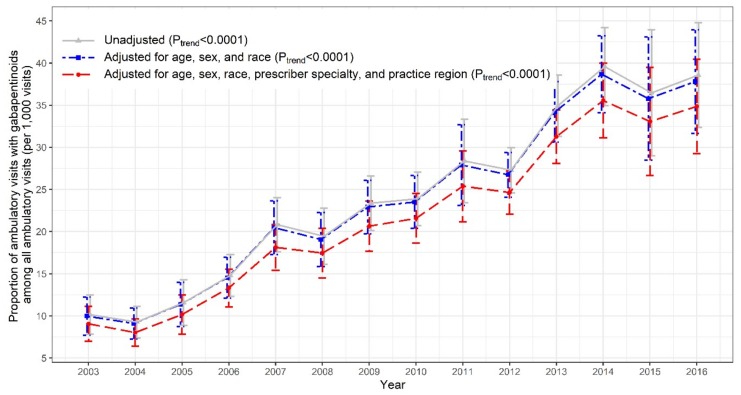
Trends in proportion of ambulatory visits involving gabapentinoids among all US ambulatory care visits: 2003–2016 National Ambulatory Medical Care Survey (NAMCS).

As shown in Figure 1, there was a substantial increase in US ambulatory care visits involving prescription gabapentinoids (7.4 to 27.0 million), opioids (41.0 to 73.0 million), and benzodiazepines (27.9 to 49.1 million) from 2003 to 2016. The trend of the adjusted proportions of gabapentinoid-involved visits among all adult ambulatory visits nearly quadrupled from 2003 to 2016 (9.1 to 34.9 per 1000 visits; *P_trend_* < 0.0001; Figure 2). The increasing trend in gabapentinoid-involved visits was mainly driven by gabapentin, while pregabalin use remained stable over time (Figures S1–S3).

Nearly half of the gabapentinoid-involved visits had concurrent use with opioids (32.9%) or benzodiazepine (15.3%). Two-thirds of gabapentinoids were prescribed by primary care physicians (45.8%), neurologists (8.2%), surgeons (6.2%), and psychiatrists (4.8%). For nearly all of the gabapentinoid-involved visits (96.6%), the reporting physician did not include an indication for a gabapentinoid among their first three diagnoses. This was higher for gabapentin than for pregabalin (98.3% vs. 89.9%). Among the visits that lacked an indication for a gabapentinoid in the first three diagnoses, the most common diagnoses were musculoskeletal system diseases (18.6%), followed by diseases of the nervous system and sense organs (12.9%), mental disorders (8.2%), and diabetes mellitus (4.5%). Over 80% of the gabapentinoid-involved visits were continuous use.

Stratification analyses showed that ambulatory care gabapentinoid-involved visits were more likely to occur among patients aged ≥65 years, who smoked, had concurrent opioid or benzodiazepine use, had two or more chronic conditions, and had governmental insurance (Figures S4–S6). Ambulatory care visits from individuals visiting neurologists and physicians located in the southern area of the US were more likely to receive gabapentinoids (Figure S6). The sensitivity analysis which included all the medications (up to 30 medications) recorded in the NAMCS resulted in 33.7 million gabapentinoid visits in 2016, compared to 27.0 million gabapentinoid visits in the primary analysis (Figure S7).

## 4. Discussion

Using NAMCS data, our study found that gabapentinoid-involved visits nearly quadrupled in the US ambulatory care settings from 2003 to 2016 from 9.1 to 34.9 per 1000 visits (primarily driven by gabapentin). Similar to Johansen’s MEPS study [4], gabapentinoid-involved visits were more likely to be from individuals less than 65 years of age and female. Half of the gabapentinoid visits had concurrent opioid and/or benzodiazepine use. Notably, we identified other characteristics associated with gabapentinoid use: white race, having governmental insurance, having multiple chronic conditions, being seen by a primary care physician, being seen by a physician practice located in the south, and being in a metropolitan area of the US.

Gabapentinoid use without a primary diagnosis for a gabapentinoid approved indication was common. While the first three diagnoses reported for the clinical visits for a patient in the NAMCS logically will not always be for a gabapentinoid related diagnosis, we expected to see a proportion of the visits sizeable enough to suggest monitoring of these patients’ symptoms and gabapentinoid use. Because of the safety concerns of misuse and abuse of gabapentinoids, this raises concerns about the appropriate health care delivery for these patients in terms of clinical management and monitoring of their gabapentinoid use. Other analyses have considered the lack of a diagnosis matching an approved indication of a gabapentinoid as a measure of off-label use [5,13]. In our analysis, the visits lacking a gabapentinoid approved indication were primarily those with musculoskeletal diseases, neuropathic pain, mental disorders, and diabetes, which align with common off-label uses [14]. If the use captured in these data is off-label use, it suggests that the physicians sampled almost always prescribed gabapentinoids in a way that is not federally approved. There are precedents for this interpretation. Radley et al. reported that 83% of gabapentin prescriptions were for off-label use in 2001 [13]. Gabapentinoids, including gabapentin and pregabalin, have been increasingly used for various types of off-label pain conditions, despite limited evidence supporting such use [15]. Restrictions on opioid prescribing, perceptions of less addiction liability and safer profiles of gabapentinoids, and inappropriate marketing may have contributed to the increasing trend in gabapentinoid use in the US in the past decade [16].

Current initiatives implemented in the US health systems to reduce gabapentinoid use include prior authorization and step therapy for pregabalin, mandatory reporting of gabapentin use to prescription drug monitoring programs in some states (e.g., Massachusetts, Ohio, and Virginia), and classifying gabapentin as a Schedule V Controlled Substance with prescribing quantity limits (Kentucky) [17,18]. However, definitions for high-risk gabapentinoid use vary [19,20,21]. While gabapentinoids are promoted as a key constituent of multimodal analgesia to reduce the opioid dosages in perioperative and other acute pain settings [22], our findings underscore the importance of safety evaluations of gabapentinoids and concomitant use of opioids/benzodiazepines and gabapentinoids in the US ambulatory care settings, especially among the largest proportion of gabapentinoid prescribers, primary care physicians. Clinicians should regularly assess the needs for continuous use of gabapentinoids, especially when the usage is for an indication that is not approved by the FDA.

Our visit-level analyses had several limitations. First, we only used eight medications to identify medications of interest, which may underestimate gabapentinoid use. We evaluated the first three provider-reported ICD-9-CM and ICD-10-CM codes in NAMCS data to determine if the visits were associated with a diagnosis for an approved gabapentinoid indication. This may overestimate the number of visits without a diagnosis for a gabapentinoid approved indication. Second, NAMCS lacks medication duration and dose information. Third, our findings represent visit-level rather than patient-level data.

Nonetheless, the increases in gabapentinoid use and variations of gabapentinoid use in clinic visits with different patient and prescriber characteristics highlight the need for additional understanding of effectiveness and safety of gabapentinoid use and the need for routine monitoring systems for individuals at high risk of misuse and abuse of gabapentinoids.

## Figures and Tables

**Table 1:** Patient and prescriber characteristics and use of gabapentinoids in US ambulatory care settings: 2003 to 2016.

Overall Weighted Estimate Visits	Gabapentinoids ^a^	Gabapentin ^a^	Pregabalin ^a^	
	260.1 Million	208.9 Million	53.7 Million	
Patient Characteristics	Weighted. %	Weighted. %	Weighted. %	SMD ^b^
≥65 years	38.2	38.8	35.0	0.08
Female	61.9	61.7	63.0	0.03
Race/ethnicity				0.04
White	85.5	85.3	86.6	
Non-white ^c^	14.5	14.7	13.4	
Current smoker	16.6	16.5	16.7	0.001
Insurance coverage ^d^				0.19
Governmental	52.3	53.8	46.7	
Commercial	38.2	36.5	45.5	
Others	4.7	4.9	3.8	
Major visit reason due to chronic problems ^e^	62.6	62.3	62.3	0.01
Concurrent use with				
Opioids	32.9	31.4	38.4	0.18
Benzodiazepines	15.3	15.6	13.6	0.06
Both opioids and benzodiazepines	6.1	5.9	6.6	0.03
≥2 chronic conditions ^d,e^	61.3	62.4	57.8	0.08
**Prescriber Characteristics**				
Specialty				0.21
Primary care	45.8	45.8	45.1	
Neurology	8.2	8.4	8.6	
Surgery	6.2	5.7	7.8	
Psychiatry	4.8	5.5	1.9	
Others	35.0	34.6	36.6	
Geographic region				0.20
Northeast	15.9	16.4	14.8	
Midwest	24.4	21.2	21.8	
South	40.6	39.0	46.9	
West	22.1	23.4	16.5	
Metropolitan area	86.8	87.1	86.1	0.03
Unapproved use ^f^	96.6	98.3	89.9	0.37

Abbreviations: SMD, standardized mean difference. ^a^ National estimates of ambulatory visits involving gabapentinoids, gabapentin, and pregabalin accounted for 2.4%, 1.9%, and 0.5% of all adult ambulatory visits, respectively. ^b^ SMD > 0.1 was considered as having non-negligible differences. ^c^ Racial groups other than White and African American only accounted for 3.0% of all gabapentinoid visits and were combined with non-white. ^d^ The percentage of missing data for insurance coverage and major visit reason due to chronic problems was 4.8% and 1.4%, respectively, from 2003 to 2016. The percentage of missing data for the variable of ≥2 chronic conditions was 1.2% from 2005 to 2016. ^e^ The number of chronic conditions was available starting in 2005. The weighted proportions were calculated based on the overall gabapentinoid visits from 2005 to 2016 (245.9 million). ^f^ An unapproved use was defined as a visit involving gabapentinoids without an FDA-approved indication for gabapentinoids among the first three physician reported diagnoses.

## Supplementary Materials

The following are available online at https://www.mdpi.com/2077-0383/9/1/83/s1, Table S1: Multum Lexicon Plus^®^ generic codes used to identify gabapentinoids, opioids, and benzodiazepines; Table S2: ICD-9-CM/ICD-10-CM codes used to identify FDA-approved indications for gabapentinoids, Figure S1: Trends in use of gabapentinoids by gabapentin vs. pregabalin in the US ambulatory settings: 2003–2016 National Ambulatory Medical Care Survey (NAMCS), Figure S2: Trends in proportion of ambulatory care visits with gabapentin use among all US ambulatory care visits: 2003–2016 National Ambulatory Medical Care Survey (NAMCS), Figure S3: Trends in proportion of ambulatory care visits with pregabalin use among all US ambulatory care visits: 2003–2016 National Ambulatory Medical Care Survey (NAMCS), Figure S4: Trends in proportion of ambulatory care visits with gabapentinoid use, stratified by age, sex, race/ethnicity, and smoking status: 2003–2016 National Ambulatory Medical Care Survey (NAMCS), Figure S5: Trends in proportion of ambulatory care visits with gabapentinoid use, stratified by concurrent use of opioids and benzodiazepines and number of chronic conditions: 2003–2016 National Ambulatory Medical Care Survey (NAMCS), Figure S6: Trends in proportion of ambulatory care visits with gabapentinoid use, stratified by insured status, major visit reason, and physician specialty and geographic region of practice location: 2003–2016 National Ambulatory Medical Care Survey (NAMCS), Figure S7: Trends in use of gabapentinoids in US ambulatory care settings, by including first eight medications and all medications available each year: 2003–2016 National Ambulatory Medical Care Survey (NAMCS).
