# Supplementary material for: Trends, Patient and Prescriber Characteristics in Gabapentinoid Use in a Sample of United States Ambulatory Care Visits from 2003 to 2016
Source: J Clin Med. 2019 Dec 29;9(1):83. doi: 10.3390/jcm9010083 (PMC7019734; doi:10.3390/jcm9010083)

Zhou L et al. Gabapentinoid Use among US Ambulatory Care Visits

Online Supplements

Table S1. Multum Lexicon Plus® Generic Codes Used to Identify Gabapentinoids, Opioids, and Benzodiazepines

Table S2. ICD-9-CM/ICD-10-CM Codes Used to Identify FDA-Approved Indications for Gabapentinoids

Figure S1. Trends in Use of Gabapentinoids by Gabapentin vs. Pregabalin in the US Ambulatory Settings: 2003-2016 National Ambulatory Medical Care Survey (NAMCS)

Deleted: ,

Deleted: , and

Figure S2. Trends in Proportion of Ambulatory Care Visits with Gabapentin Use among all US Ambulatory Care Visits: 2003-2016 National Ambulatory Medical Care Survey (NAMCS)

Figure S3. Trends in Proportion of Ambulatory Care Visits with Pregabalin Use among all US Ambulatory Care Visits: 2003-2016 National Ambulatory Medical Care Survey (NAMCS)

Figure S4. Trends in Proportion of Ambulatory Care Visits with Gabapentinoid Use, Stratified by Age, Sex, Race/Ethnicity, and Smoking Status: 2003-2016 National Ambulatory Medical Care Survey (NAMCS)

Figure S5. Trends in Proportion of Ambulatory Care Visits with Gabapentinoid Use, Stratified by Concurrent Use of Opioids and Benzodiazepines and Number of Chronic Conditions: 2003-2016 National Ambulatory Medical Care Survey (NAMCS)

Figure S6. Trends in Proportion of Ambulatory Care Visits with Gabapentinoid Use, Stratified by Insured Status, Major Visit Reason, and Physician Specialty and Geographic Region of Practice Location: 2003-2016 National Ambulatory Medical Care Survey (NAMCS)

Figure S7. Trends in Use of Gabapentinoids in US Ambulatory Care Settings, by Including First Eight Medications and All Medications Available Each Year: 2003-2016 National Ambulatory Medical Care Survey (NAMCS)

Table S1. Multum Lexicon Plus® Generic Codes Used to Identify Gabapentinoids, Opioids, and Benzodiazepines

Medication class and drugs	Multum's Generic codes
**Gabapentinoids**	
Gabapentin	d03182
Pregabalin	d05508
**Opioids**	
Buprenorphine	d00840, d04819
Butorphanol	d00838
Codeine	a11076, d00012, d03357, d03364, d03393, d03394, d03398, d03423, d03426, n08029
Dihydrocodeine	d03168
Fentanyl	d00233
Hydrocodone	a10897, a10956, a11768, d03075, d03340, d03356, d03361, d03396, d03428, d03915, d04225
Hydromorphone	d00255
Meperidine	d00017, d03433
Methadone	d00050
Morphine	d00308
Oxycodone	d00329, d03431, d03432
Oxymorphone	d00833
Pentazocine	d00334
Propoxyphene	d00360, d03434
Tramadol	d03826, d04766
**Benzodiazepines**	
Alprazolam	d00168
Chlordiazepoxide	d00189, d03462, d03492
Clonazepam	d00197
Clorazepate	d00198
Diazepam	d00148
Estazolam	d00915
Flurazepam	d00238
Halazepam	d00904
Lorazepam	d00149, n15002
Midazolam	d00301
Oxazepam	d00040
Prazepam	a54760
Quazepam	d00917
Temazepam	d00384
Triazolam	d00397

Table S2. ICD-9-CM and ICD-10-CM Codes Used to Identify FDA-Approved Indications for Gabapentinoids

Diseases/Conditions	ICD-9-CM codes	ICD-10-CM codes
Partial seizures	780.31, 780.32, 780.33, 780.39	R56.00, R56.01, R56.1, R56.9
Postherpetic neuralgia	053.10, 053.11, 053.12, 053.13, 053.14, 053.19	B02.21, B02.22, B02.23, B02.24, B02.29
Restless legs syndrome (gabapentin only)	333.94	G25.81
Diabetic peripheral neuropathy (pregabalin only)	250.60, 250.61, 250.62, 250.63, 357.2	E10.4x, E11,4x, E13.4x
Fibromyalgia (pregabalin only)	729.1	M60.9, M79.1, M79.7
Neuropathic pain associated with spinal cord injury (pregabalin only)	344.0x, 344.1x, 344.6x, 806.xx, 952.xx (spinal cord injury) with 338.0 (central pain syndrome)	G82.xx, G83.4, S14.1x, S14.2x, S24.1x, S24.2x, S34.0x, S34.1x (spinal cord injury) with G89.0

Zhou L et al. Gabapentinoid Use among US Ambulatory Care Visits

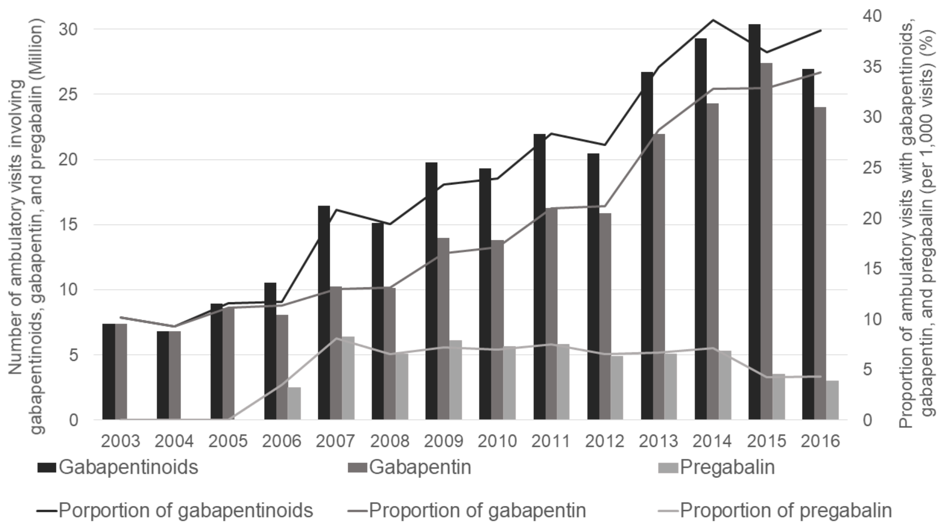

Figure S1. Trends in Use of Gabapentinoids by Gabapentin vs. Pregabalin in the US Ambulatory Settings: 2003-2016 National Ambulatory Medical Care Survey (NAMCS)

Deleted: ,

Deleted: , and

National estimates of ambulatory visits involving pregabalin in 2005 was not reported, based on NAMCS's recommendation, due to the unweighted number less than 30, which yielded unreliable national estimates.

Zhou L et al. Gabapentinoid Use among US Ambulatory Care Visits

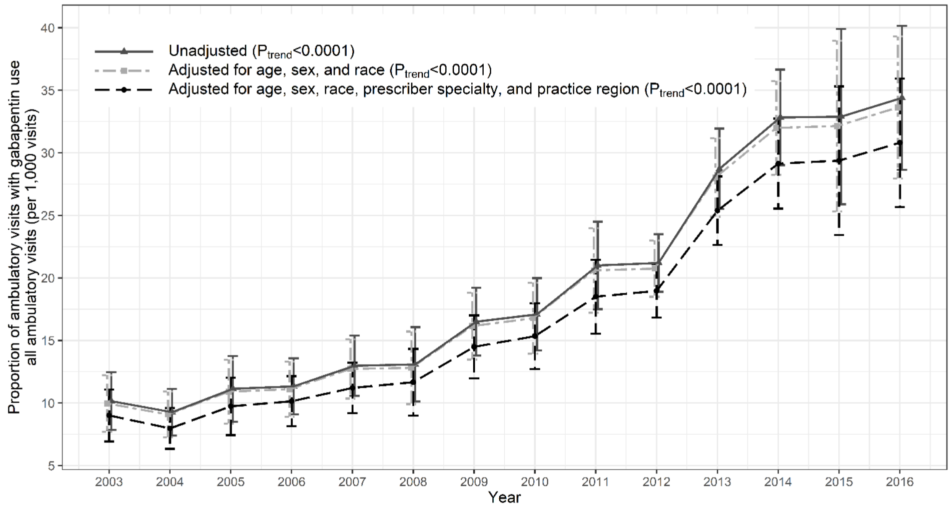

Figure S2. Trends in Proportion of Ambulatory Care Visits with Gabapentin Use among all US Ambulatory Care Visits: 2003-2016 National Ambulatory Medical Care Survey (NAMCS)

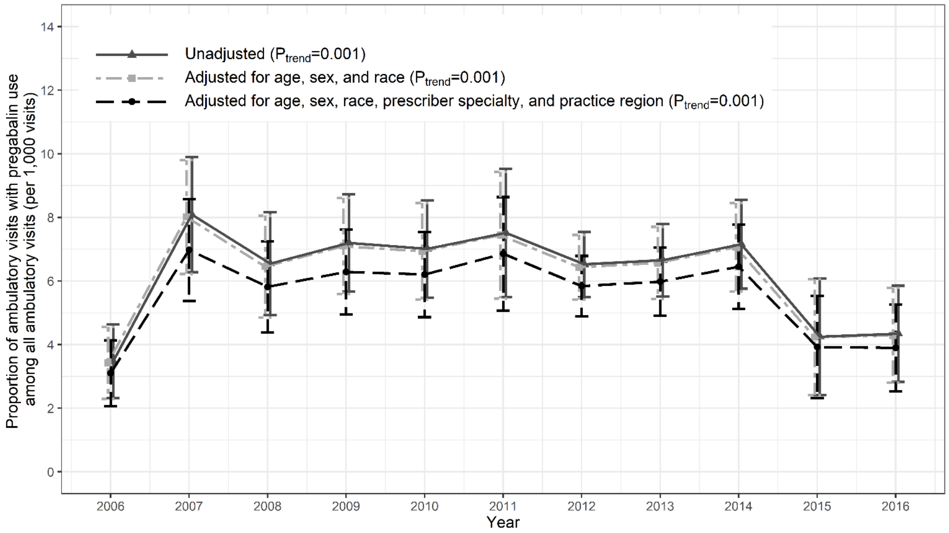

Figure S3. Trends in Proportion of Ambulatory Care Visits with Pregabalin Use among all US Ambulatory Care Visits: 2003-2016 National Ambulatory Medical Care Survey (NAMCS)

When only including the data from 2007 to 2014, the P_{trend} for the unadjusted model, for the model adjusted for age, sex, and race, and the model adjusted for age, sex, race, prescriber specialty, and practice region were 0.78, 0.76, and 0.83, respectively.

Zhou L et al. Gabapentinoid Use among US Ambulatory Care Visits

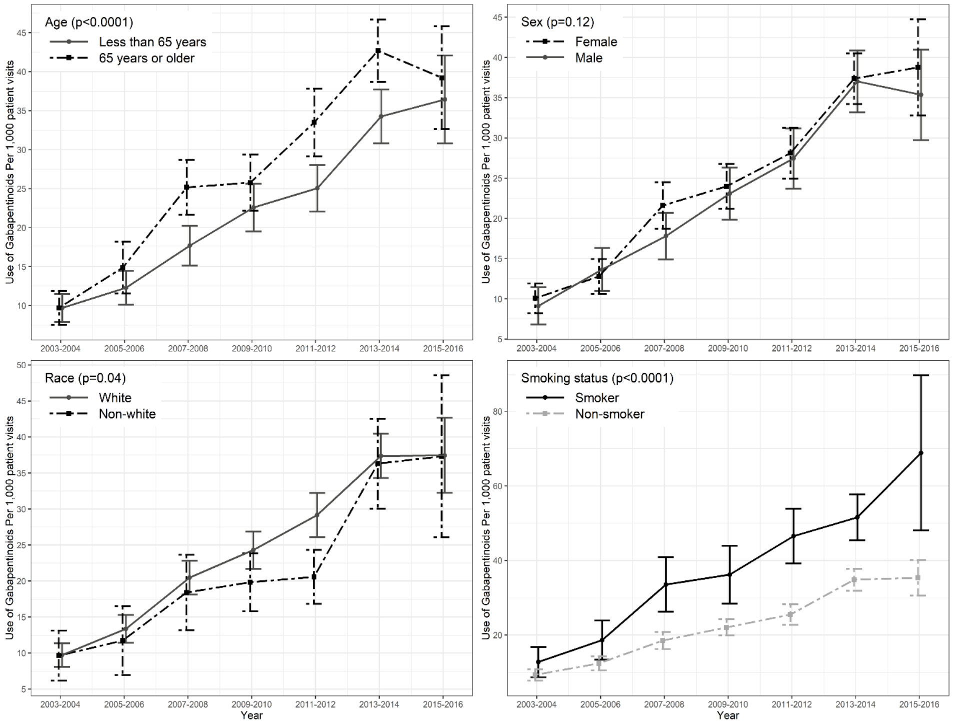

Figure S4. Trends in Proportion of Ambulatory Care Visits with Gabapentinoid Use, Stratified by Age, Sex, Race/Ethnicity, and Smoking Status: 2003-2016 National Ambulatory Medical Care Survey (NAMCS)

Zhou L et al. Gabapentinoid Use among US Ambulatory Care Visits

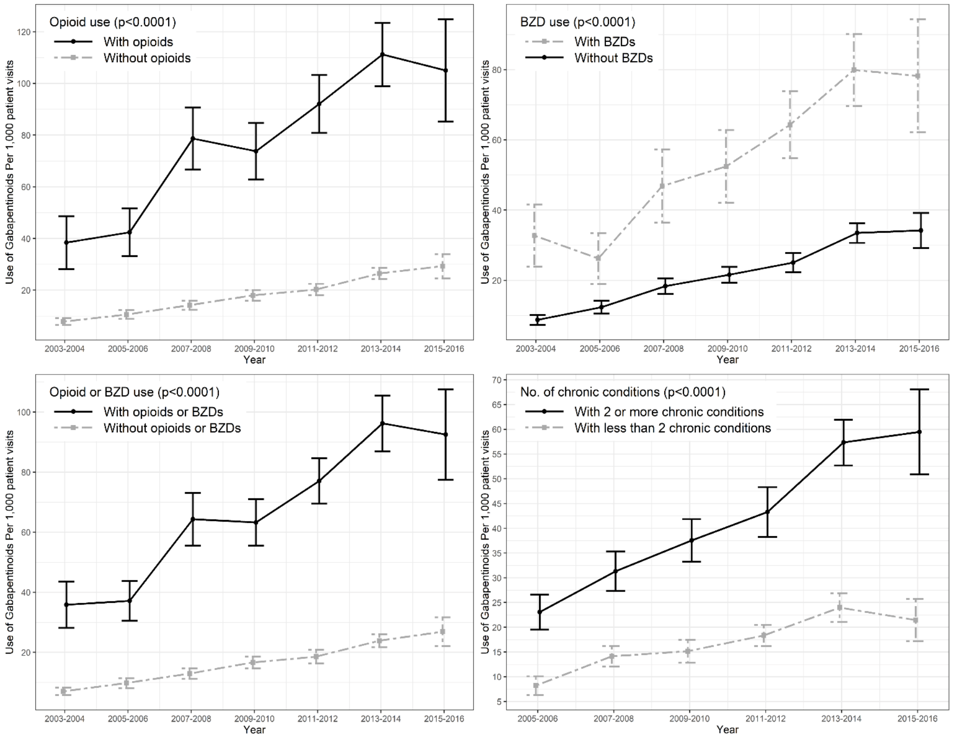

Figure S5. Trends in Proportion of Ambulatory Care Visits with Gabapentinoid Use, Stratified by Concurrent Use of Opioids and Benzodiazepines and Number of Chronic Conditions: 2003-2016 National Ambulatory Medical Care Survey (NAMCS)

Abbreviations: BZDs: benzodiazepines

Formatted: Font: 11 pt, Not Bold

Zhou L et al. Gabapentinoid Use among US Ambulatory Care Visits

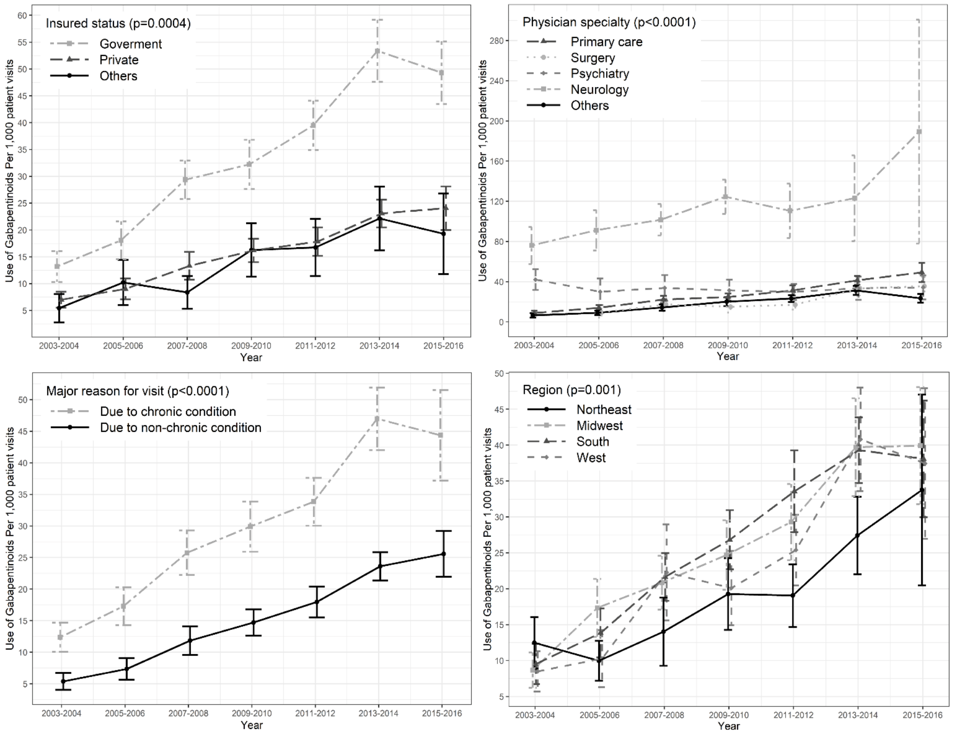

Figure S6. Trends in Proportion of Ambulatory Care Visits with Gabapentinoid Use, Stratified by Insured Status, Major Visit Reason, and Physician Specialty and Geographic Region of Practice Location: 2003-2016 National Ambulatory Medical Care Survey (NAMCS)

National estimates of gabapentinoid visits prescribed by surgeons between 2003 and 2014 were not reported because the unweighted number was less than 30.

Zhou L et al. Gabapentinoid Use among US Ambulatory Care Visits

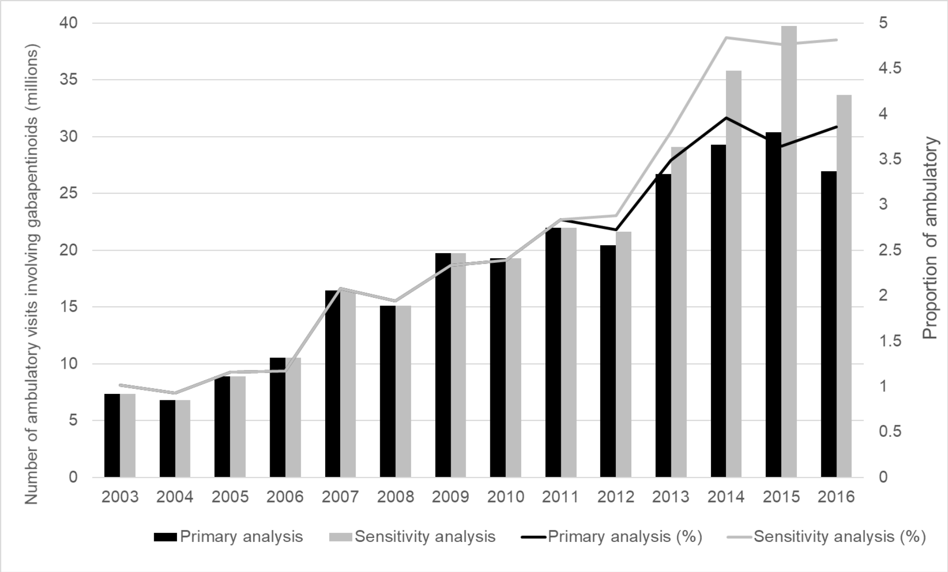

Figure S7. Trends in Use of Gabapentinoids in US Ambulatory Care Settings, by Including First Eight Medications and All Medications Available Each Year: 2003-2016 National Ambulatory Medical Care Survey (NAMCS)

Deleted:

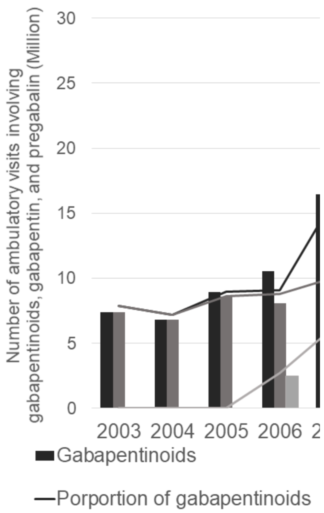

Supplement: Supplementary file 1 [file jcm-09-00083-s001.zip › jcm-665627_Trends in gaba use in NAMCS_supplement_RV/jcm-665627_Trends in gaba use in NAMCS_supplement_RV.pdf]
